# Impact of BMI and smoking in adolescence and at the start of pregnancy on birth weight

**DOI:** 10.1186/s12884-023-05529-1

**Published:** 2023-03-27

**Authors:** Rebecka Bramsved, Staffan Mårild, Maria Bygdell, Jenny M. Kindblom, Ingela Lindh

**Affiliations:** 1 Department of Pediatrics, Institute of Clinical Sciences, Sahlgrenska Academy at Gothenburg University, Gothenburg, Sweden; 2 Centre for Bone and Arthritis Research, Department of Internal Medicine and Clinical Nutrition, Institute of Medicine, Sahlgrenska Academy at Gothenburg University, Gothenburg, Sweden; 3 Region Västra Götaland, Sahlgrenska University Hospital, Pediatric Clinical Research Center, Gothenburg, Sweden; 4 Department of Obstetrics and Gynecology, Institute of Clinical Sciences, Sahlgrenska Academy at Gothenburg University, Gothenburg, Sweden

**Keywords:** Birth weight, Body mass index, Overweight, Obesity, Pregnancy, Adolescence, Smoking

## Abstract

**Background:**

Birth weight is an indicator of intra-uterine conditions but also a determinant for future health. The importance of preconception health for a healthy birth weight has been emphasized, but evidence is lacking on how modifiable factors in adolescence, such as body mass index (BMI) and smoking, affect future pregnancy outcome. We evaluated associations between BMI and smoking in adolescence and at the start of pregnancy and birth weight of the first-born child.

**Methods:**

This longitudinal study included 1256 mothers, born 1962–1992, and their first-born children, born between 1982–2016. Self-reported questionnaire information on weight, height and smoking at age 19 was cross-linked with national register data obtained at the start of pregnancy and with the birth weights of the children. Univariable and multivariable linear regressions were performed to determine the impact of maternal factors at 19 years of age and at the start of the pregnancy respectively, and the importance of BMI status at these points of time for the birth weight of the first child.

**Results:**

BMI and smoking at the start of the pregnancy displayed strong associations with birth weight in a multivariable analysis, BMI with a positive association of 14.9 g per BMI unit (95% CI 6.0; 23.8 *p* = 0.001) and smoking with a negative association of 180.5 g (95% CI -275.7; -85.4) *p* = 0.0002). Smoking and BMI at 19 years of age did not show this association. Maternal birth weight showed significant associations in models at both time-points. Becoming overweight between age 19 and the start of the pregnancy was associated with a significantly higher birth weight (144.6 (95% CI 70.7;218.5) *p* = 0.0002) compared to mothers with normal weight at both time points.

**Conclusions:**

Our findings indicate that the time period between adolescence and first pregnancy could be a window of opportunity for targeted health promotion to prevent intergenerational transmission of obesity.

## Background

The global disease burden of overweight and obesity is now one of the greatest threats to public health worldwide [1] and has proven difficult to treat, despite great efforts and vast costs. Especially worrying is the increasing prevalence of obesity among women of reproductive age [2]. Women entering pregnancy with obesity have a higher risk of a wide range of adverse pregnancy and delivery complications. The effects of maternal obesity further extends to the next generation, creating an intergenerational cycle of obesity, with birthweight as a mediator [3, 4]. Life-style interventions in overweight women during pregnancy have not been successful in reducing the frequency of macrosomia [5, 6] and a meta-analysis concluded that weight loss prior to pregnancy is probably needed to achieve optimum pregnancy outcomes [7].

Smoking during pregnancy is another well-known modifiable risk factor that has been linked to reduced birth weight in several large studies [8, 9]. Smoking not only affects birth weight, but also linear growth and head circumference [10]. The negative effect of smoking extends into childhood and has been shown to increase the risk of childhood overweight [11]. As for overweight, interventions to curb the negative effects of smoking during pregnancy have not shown to be sufficient when initiated after conception [12].

Life course epidemiology is a useful technique capable of examining preconceptional factors and their effects on maternal and child health as it considers the timing and duration of exposures and their potential latent or cumulative effects. Adolescence could in this perspective be regarded as a sensitive period for future pregnancy outcome, since unhealthy life-style behaviors such as smoking and poor diet often originate during the teenage years [13]. There is, however, very little knowledge regarding the association between BMI and smoking in adolescence and subsequent pregnancy outcome. The longitudinal study of 19-year-old women in Gothenburg offers a unique possibility to address this knowledge gap.

The aim of this study was to evaluate the association between these modifiable maternal factors, BMI and smoking in the period between adolescence and young adulthood with the birth weight of the first-born child. We hypothesized that the adolescent period is a window of opportunity for improving future pregnancy outcome and the health of the next generation.

## Methods

### Study population and data sources

**Fig. 1:**
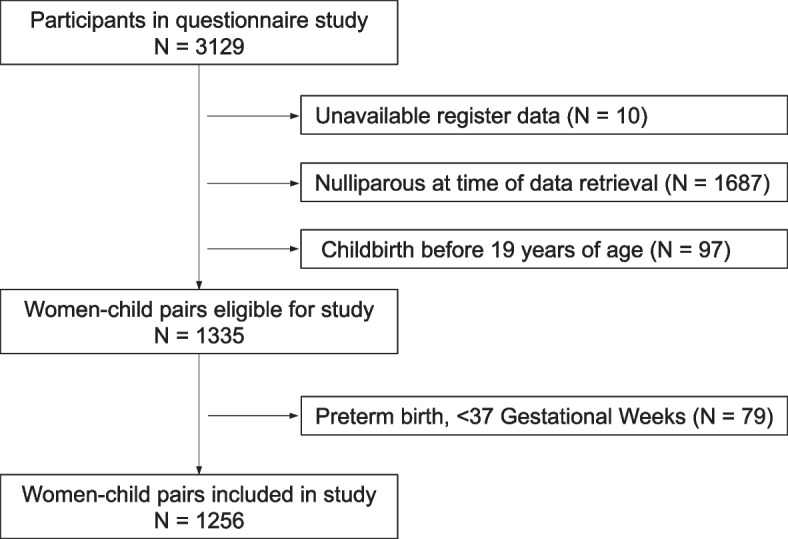
Flowchart of inclusion and exclusion criteria for the study population

This study originates from the prospective longitudinal study of 19-year-old women in Gothenburg, Sweden, initiated in 1981 [14]. The participants were randomly identified from the population register and were invited at age 19 in 1981 (born 1962), in 1991 (born 1972), in 2001 (born 1982) and in 2011 (born 1992) as previously described [15]. Questionnaires regarding weight, height, smoking and reproductive health were sent out by mail at the study start and subsequently every fifth year. Validation of the questionnaires was performed and is described in detail in a previous publication [16]. No changes were made in the questionnaires between study start and later on, except for the addition of newly available contraceptives among the answer options. For the purpose of the present study, all women who had given birth to at least one child after the age of 19 years at the time of data retrieval were eligible. The first-born children were born between 1982 and 2016. To avoid confounding due to prematurity, children born before 37 weeks of gestation were excluded. One child per twin pair was included. A flow chart of the inclusion process is shown in Fig. 1.

Auxiliary data to the information from the questionnaires regarding the women and their first-born children were retrieved by linkage to Swedish national registers, using the unique personal identification number allocated to every Swedish citizen [17]. The Medical Birth Register (MBR) at the National Board of Health and Welfare [18] was initiated in 1973 for surveillance of prenatal, obstetric and neonatal care, as well as for research. High quality data on all deliveries are registered, as well as information from the start of pregnancy, usually at about 8 weeks of pregnancy. Self-reported information on smoking, alcohol consumption, occupation and family situation is obtained, and a midwife measures weight [19]. For women born in 1962 and 1972, birth weights were retrieved from archived child and school health records since the Swedish MBR had not been initiated at the time these women were born. Information on education, income and country of birth was retrieved from the Longitudinal Integrated Database for Health Insurance and Labor Market Studies (LISA) [20] and Total Population Register [21] at Statistics Sweden for the year before the birth of the first child.

### Exposure variables

The main exposure variables were maternal birth weight together with weight status at 19 years of age and at the start of the pregnancy and smoking status both at 19 years age and at the start of the pregnancy. Self-reported information at 19 years of age and register derived information from the first visit to the antenatal clinic (defined as start of pregnancy) on weight and height were used to calculate BMI (weight in kg/height in meter^2^) and categorized into underweight (BMI < 18.5), normal weight (BMI 18.5–24.9), overweight (BMI 25–29.9) or obesity (BMI >  = 30) [22]. Women who reported smoking at least 1 cigarette daily were categorized as smokers. Smoking at start of pregnancy was defined as reporting daily smoking at either 3 months before start of pregnancy or at the first visit to the maternal health clinic at 8 weeks of pregnancy. Maternal age at start of pregnancy was analyzed as a continuous variable. Education was categorized as low (0–12 years) or high (> 12 years). To assess income in the study population, the individual disposable income defined by Statistics Sweden was retrieved for the year before the birth of the first child. Income was index-related to the year 2015 and categorized as above (“high”) or below (“low”) the median income for the study population. Birth country of the parents of the women participating in the study was obtained from LISA at Statistics Sweden and categorized as Swedish born if both parents were born in Sweden. Children were categorized as small for gestational age (SGA) if having a birth weight below minus two standard deviations for their gestational age, and large for gestational age (LGA) if birth weight was above two standard deviations for their gestational age.

### Statistical analyses

Descriptive data are presented with mean and standard deviation and categorical variables were presented with number and percentage. In order to test changes between 19 years of age and the start of the first pregnancy Wilcoxon sign rank test was used for continuous variables and Sign test for dichotomous and ordered categorical variables.

In order to evaluate predictors of birth weight, univariable linear regressions were performed with the child’s birth weight as a dependent variable and maternal birth weight, mothers’ parents’ country of birth, maternal weight, height, BMI and smoking at 19 years as well as maternal age, weight, BMI, smoking, education and income at the start of the first pregnancy as predictors. BMI and smoking at 19 years of age and at the start of the first pregnancy were analyzed in a multivariable regression analysis, adjusted for maternal birth weight and maternal age and year of birth, and in the next step further adjusted for gestational age and sex of the child. Regression analysis was also performed based on groups of change in BMI status between 19 years of age and the start of pregnancy. This analysis was adjusted for maternal age at the first pregnancy, maternal birth year, maternal smoking at the start of pregnancy as well as gestational week and sex of the child. Beta (95% CI), standardized beta (95% CI) (inn the univariable analyses), *p*-value, and R2 are presented from the regression analyses. The standardized beta was calculated as beta*s_x_/s_y_, where s_x_ and s_y_ are the (estimated) standard deviations of x and y, respectively. Interaction testing for sex of the child and exposure variables were performed with a significance level set to 0.2. All analyses were performed using SAS 9.4 SAS Institute Inc., Cary NC, USA. All testing was made using a with alpha level 0.05 (interactions 0.20) with two-sided t-tests.

## Results

### Characteristics of the study population

We included 1256 women-child pairs in the study (Fig. 1). Table 1 depicts the maternal characteristics at birth, at 19 years of age and at the start of pregnancy. The mean age at the start of the first pregnancy was 27.6 years (SD 4.6), min 20 years, max 40 years of age. Between 19 years of age and the start of the pregnancy, the mean maternal weight increased from 59.9 kg (SD 9.1) to 65.1 kg (SD 11.0). The prevalence of overweight and obesity increased accordingly during this time period, from 6.3% to 20.0% for overweight and from 1.4% to 5.3% for obesity. The number of smokers decreased, from 35.8% at 19 years of age to 14.7% at the start of the first pregnancy (*p* < 0.0001 for all comparisons). Regarding diagnoses that potentially influence birth weight, 6 women (0.5%) had hypertensive disorders and 9 women (0.7%) had diabetes in any form. No exclusions or adjustments were performed for these diagnoses. Table 2 shows the characteristics of the children at birth.

**Table 1:** Characteristics of the mothers (*n* = 1256) in the study cohort

**Variables**	**Mean (SD)/percentage**		**n**	
**Birth weight** (kg)	3.46 (0.52)		1047	
**Birth length** (cm)	50.1 (2.3)		706	
**Age at start of first pregnancy**	27.6 (4.6)		1256	
**Disposable Income**^a^	167 200 (85 500)		1256	
Low (< median 146 800)	49.9%		627	
High (> median 146 800)	50.1%		629	
**Education level**^**b**^			1227	
Low (0–12 years)	58.6%		719	
High (> 12 years)	41.4%		508	
**Parental Country of Birth**			1223	
Sweden	72.5%		887	
Other	27.5%		336	
	**19 years**	**Pregnancy start**		
**Weight** (kg) mean (SD)	59.9 (9.1)	65.1 (11.0)	1227	1030
**Height** (cm) mean (SD)	167.1 (6.0)		1245	
**BMI** (kg/m^2^) mean (SD)	21.4 (2.8)	23.3 (3.6)	1225	1030
Underweight (< 18.5)	7.9%	4.8%	97	49
Normal (18.5–24.9)	84.4%	69.9%	1043	720
Overweight (25–29.9)	6.3%	20.0%	77	206
Obese (> 30)	1.4%	5.3%	17	55
**Smoking status**			1233	1181
Smoker	35.8%	14.7%	441	174

^a^ At the start of the first pregnancy. The sum of incomes and benefits minus taxes and negative

transfers, per year, in Swedish crowns. Index-related to the year of 2015

^b^At the start of the first pregnancy

**Table 2:** Characteristics of the children (*n* = 1256) in the study cohort

**Variables**	**Mean (SD)/percentage**	**n**
**Birth weight** (kg)	3.51 (0.48)	1256
**Birth length** (cm)	50.4 (2.2)	1246
**Boys**	52.3%	657
**Girls**	47.7%	599
**Twins**^a^	1%	12
**Large for gestational age (LGA)**	1.8%	22
**Small for gestional age (SGA)**	3.4%	42

^a^ One child per twin pair included

### Associations between maternal factors and infant birth weight

Since birth weight differs by sex of the child, we tested whether there was an interaction between sex and the exposure factors in the association with birth weight. There was no significant interaction with sex of the child (*p* > 0.2) and hence results are presented for girls and boys together.

**Fig. 2:**
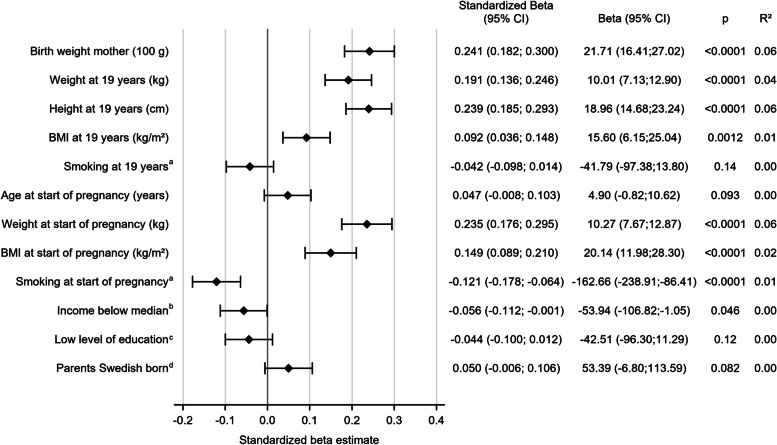
Associations between maternal factors and birth weight of first-born child. Results of univariable analyses presented as standardized beta (95 % CI) and beta (95 % CI) The Beta-column show the effect on birth-weight (in gram) for each unit change given for maternal features in the left-hand column ^a^Smoking categorized as smoker or non-smoker ^b^Disposable income for the year before birth of the first child, income below median of the group 146 800 Swedish Crowns, index related to the year of 2015 ^c^0-12 years of education ^d^Both parents of the study participant born in Sweden

Univariable linear regressions, shown in Fig. 2, demonstrated significant positive associations of infant birth weight with maternal BMI at both 19 years of age and at the start of the pregnancy. Smoking at 19 years was not significantly associated with birth weight, while smoking at the start of pregnancy was associated with a reduction of birth weight by 163 g (95% CI -238.9; -86.4, *p* < 0,001). Of the socioeconomic factors investigated, maternal income displayed a significant association with infant birth weight, with a reduction in birth weight of 54 g for income below the median (95% CI -106.8; -1.1 *p* = 0.046). The highest R^2^ was seen for maternal birth weight, height at 19 years age and weight at the start of pregnancy, each explaining 6% of the variance in infant birth weight (R2 0.06 *p* < 0.0001).

We evaluated the predictive value of maternal BMI and smoking at 19 years of age and at start of pregnancy in multivariable models (Table 3). The models evaluated maternal BMI, smoking and maternal birth weight effect on birth weight; Model 1A and 1B at 19 years of age and Model 2A and 2B at the start of pregnancy. All models were adjusted for maternal age at the start of the first pregnancy and birth year of mother, and models B additionally for gestational age and sex of the child. Neither maternal BMI nor smoking at 19 years of age were independently associated with birth weight of the first-born child (Table 3, Model 1A). In contrast, maternal BMI and smoking at the start of the pregnancy were both associated with birth weight (Table 3, Model 2A), BMI with a positive association of 14.9 g per BMI unit (95% CI 6.0; 26.0 *p* = 0.001) and smoking with a negative association of 180.5 g (95% CI -275.7; -85.4 *p* = 0.0002). R^2^ of this model was 0.08. Maternal birth weight was positively associated with birth weight in both models. Adjusting for gestational age and sex of the child increased the degree of explanation in the models but did not change the association with the examined exposures (Table 3, Model 1B and 2B).

**Table 3:** Prediction of birth weight of first-born child by BMI and smoking at 19 years of age and at the start of the first pregnancy

	**Model 1** **At 19 years of age, *n* = 1006**				**Model 2** **At start of first pregnancy, *n* = 846**			
	**A**^a^ *R*^2^ = 0.06		**B**^b^ *R*^2^ = 0.24		**A**^a^ *R*^2^ = 0.08		**B**^b^ *R*^2^ = 0.24	
	**Beta (95% CI)**	***p* value**	**Beta (95% CI)**	***p* value**	**Beta (95% CI)**	***p* value**	**Beta (95% CI)**	***p* value**
**Maternal Birth Weight (100 g)**	22.1 (16.6; 27.5)	< .0001	19.0 (14.0; 24.0)	< .0001	20.1 (14.1;26.0)	< .0001	19.5 (14.1;24.9)	< .0001
**BMI (kg/m**^**2**^**)**	7.0 (-4.1; 18.0)	0.22	3.2 (-6.8; 13.3)	0.53	14.9 (6.0; 23.8)	0.0010	12.1 (4.0; 20.3)	0.0033
**Smoking**	-29.9 (-89.8;30.1)	0.33	-30.3 (-84.6; 24.0)	0.27	-180.5 (-275.7; -85.4)	0.0002	-154.1 (-241.1;-67.2)	0.0005

^a^ Adjusted for maternal age and birth year

^b^ Adjusted for maternal age, birth year, gestational age and sex of child

### Impact of changes in BMI status between 19 years of age and the start of the first pregnancy on the birth weight of the first-born child

**Fig. 3:**
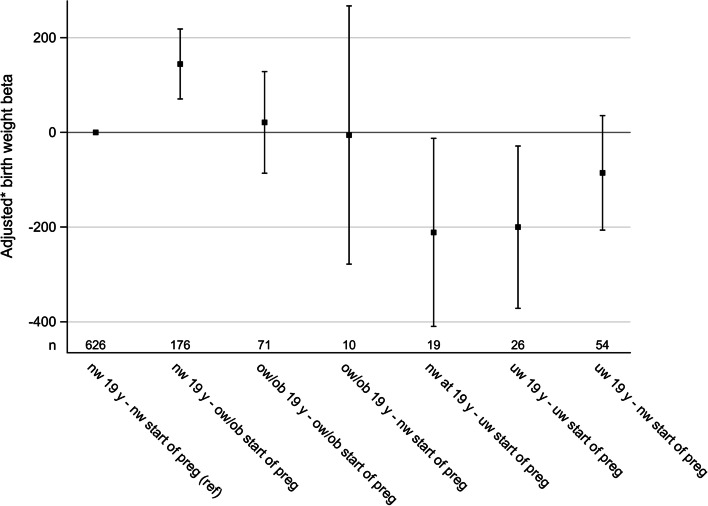
Impact of changes in BMI status from 19 years of age to the start of the first pregnancy on birth weight, results from a multivariable regression model (*n* = 982) *Beta (95 %CI) adjusted for maternal birth year, age at start of pregnancy and smoking, gestational week and sex of child The group with normal weight (BMI 18.0-24.9 kg/m^2^) at both 19 years of age and at start of pregnancy is used as reference nw – normal weight, ow/ob – overweight or obesity, uw – underweight, y – years

In multivariable regression models grouped according to change in BMI status between19 years of age and the start of the first pregnancy (shown in Fig. 3), shifting from normal weight at 19 years of age to overweight or obesity at the start of the pregnancy (*n* = 176) was associated with significantly higher birth weight of the first-born child compared to that of women with normal weight at both time points (*n* = 626), Beta 144.6 (95% CI 70.7;218.5) *p* = 0.0002.

Children born to women who were overweight or obese at both age 19 and at the start of the pregnancy (*n* = 71), did not differ in birth weight from the children of mothers in the normal weight reference group. The groups with 54, 19, 26 and 10 individuals respectively were small, with wide confidence intervals, and thus no certain conclusions can be made for these BMI groups.

## Discussion

A healthy birth weight is important for the prevention of intergenerational transmission of obesity [23]. Studies indicate that interventions during pregnancy are insufficient in their aim to normalize birth weight, highlighting the importance of preconceptional health [13]. In the present study, which included 1256 women-child pairs, we evaluated the influence of the women’s BMI and smoking in both late adolescence, at 19 years of age, and at the start of the first pregnancy on the birth weight of her first born child. To our knowledge, this is the first study evaluating BMI and smoking in late adolescence in relation to future pregnancy outcomes. We found that BMI and smoking at 19 years of age and at the start of the pregnancy differed regarding association with birth weight. The impact on birth weight of these variables in adolescence was negligible, while both factors at the start of the pregnancy were significantly associated. We hypothesized that the changes that occurred in maternal BMI or smoking from age 19 to the start of the first pregnancy may cause birth weight deviations associated with an increased risk of future disease. Both BMI and smoking changed significantly between these two time points. Mean BMI and prevalence of overweight and obesity increased, and we found that developing overweight after the age of 19 was associated with higher birth weight of the first-born child compared to children of women that maintained a normal weight. The rate of smoking, on the other hand, decreased significantly.

Initiating smoking during the adolescent years has widespread negative long-term health effects [24], and smoking cessation remains a public health priority. Our results show that there is no persistent negative effect on birth weight related to smoking in late adolescence if terminated before the start of pregnancy. These results indicate that there are great health benefits to be gained by intensifying efforts to promote smoking cessation in young women in time to avoid affecting future pregnancy, since previous studies have shown that cessation of smoking in early pregnancy is not enough to prevent the negative impact on fetal growth [12].

Maternal obesity when entering pregnancy has been shown to influence not only birth weight but also the distribution and amount of fat mass in relationship to lean mass in the neonate [25]. Maternal BMI seems to be independently associated with increased adiposity in the new-born [26] and an additive effect of high birth weight and obesity at the start of pregnancy has been observed for the risk of having a LGA child [27]. However, the importance of whether maternal overweight is present since childhood or develops after adolescence for the birth weight of their first-born child is not known. No difference was seen for birth weight of the first-born child for women who were overweight or obese at both age 19 and at the start of the pregnancy compared with women with a normal weight at both age 19 and at the start of pregnancy, in contrast to developing overweight or obesity after adolescence. We can only speculate on the reason behind this difference. A recent weight gain in a woman entering pregnancy could be associated with higher levels of IGF-1 and insulin than in a woman with stable overweight. These levels might affect body composition and the birth weight of the child and increase the risk of unhealthy weight development in childhood. However, these pathophysiological theories need to be confirmed in future studies.

Our findings support the conclusions by Hanson et al., that lifestyle interventions in pregnant women do not limit gestational weight gain to the degree required to have a meaningful impact on pregnancy outcomes. Focus for interventions should be in the preconception period [28]. Our findings suggest a window of opportunity to promote healthy birth weight development for the first child between late adolescence and pregnancy.

Socioeconomic factors, such as family income and education, are well known factors affecting the risk for development of childhood obesity. In a previous study from our group, we found parental income to be positively associated with birth weight [29]. In the present study, income was weakly associated with birth weight, while the other socioeconomic factors investigated did not show any association with birth weight. These findings suggest that socioeconomic status mainly affects childhood BMI development through later lifestyle related factors.

### Strengths and limitations

The main strengths of this study are the availability of longitudinal information for each woman on weight, height and smoking status at 19 years of age. This setting allows us to evaluate weight change and changes in smoking status during the time interval between late adolescence and at the start of pregnancy. The national Swedish registers are reliable and nearly complete [19], which allowed us to examine the effects of numerous exposures, such as smoking, maternal age, country of birth, income and education on birth weight. Information regarding these factors has been requested in previous studies on the subject of maternal overweight and birth weight of her child [4].

The limitations include a relatively low prevalence of overweight and obesity among mothers both at 19 years of age and at the start of pregnancy, which might be due to selection bias in the questionnaire study. The prevalence of birth weights outside normal range was also low, which prevented analyses of risk of LGA or SGA in the child.

## Conclusions

Obesity is the leading cause of poor health worldwide today [1, 30]. The increasing prevalence of pre-conceptional maternal obesity is especially worrying knowing that it has a negative effect on the health of the next generation [2]. There is increasing evidence suggesting that interventions during pregnancy are not sufficient to reduce the negative effect of maternal overweight [13] and attention must be directed towards better nutrition in adolescence [31].

In this study, we had the rare opportunity to study BMI and smoking habits in women both at 19 years of age and at the start of their first pregnancy. We demonstrate that smoking at age 19 is not independently associated with birth weight of the first-born child, high-lighting late adolescence as highly important for promotion of smoking cessation when striving for a healthy birth weight in the next generation. Our results further indicate that weight gain during the period between late adolescence and the first pregnancy might be of importance for infant birth weight.

## Data Availability

The datasets generated during and/or analyzed during the current study are available from the corresponding author on reasonable request.

## Abbreviations

BMI: Body Mass Index
LGA: Large for Gestational Age
LISA: Longitudinal Integrated Database for Health Insurance and Labor Market Studies
MBR: Medical Birth Register
SD: Standard Deviation
SGA: Small for Gestational Age
